# A speech therapy treatment protocol for exercise induced laryngeal obstruction

**DOI:** 10.3389/fped.2024.1356476

**Published:** 2024-06-13

**Authors:** Tom Karlsen, Kristine Vreim, Ola D. Røksund, Maria Vollsæter, Praveen Muralitharan, Thor Andre Ellingsen, John-Helge Heimdal, Thomas Halvorsen, Hege Clemm

**Affiliations:** ^1^ Department of Pediatric and Adolescent Medicine, Haukeland University Hospital, Bergen, Norway; ^2^ Stemmelogopedi AS, Private Speech Therapy Clinic, Bergen, Norway; ^3^ Faculty of Health and Social Sciences, Western Norway University of Applied Sciences, Bergen, Norway; ^4^ Department of Otolaryngology and Head and Neck Surgery, Haukeland University Hospital, Bergen, Norway; ^5^ Department of Clinical Science, University of Bergen, Bergen, Norway; ^6^ Department of Surgery, Haukeland University Hospital, Bergen, Norway; ^7^ Department of Clinical Medicine, University of Bergen, Bergen, Norway

**Keywords:** EILO, VCD, exercise, speech therapy, treatment, protocol

## Abstract

**Background:**

Exercise induced laryngeal obstruction (EILO) is a common cause of exertional breathing problems in young individuals, relevant to 5%–7% of young people. It is caused by paradoxical inspiratory adduction of laryngeal structures and diagnosed by continuous visualization of the larynx during high intensity exercise. Empirical data suggest that EILO consists of different subtypes that require different therapeutic approaches. Currently applied treatment approaches do not rest on randomized controlled trials (RCTs), and thus evidence-based guidelines cannot be established. This protocol describes the speech therapy treatment approach which is applied to EILO patients in a large prospective RCT called HelpILO.

**Methods and analysis:**

Consenting patients consecutively diagnosed with EILO at Haukeland University Hospital are randomized into four treatment arms. Speech therapy is represented in two of these, provided in a structured manner based on general speech therapy principles and abdominal breathing, combined with experience obtained with these patients at our hospital during the last decades. The main outcome measure of HelpILO is changes of laryngoscopically visualized laryngeal adduction, rated at peak exercise before vs. after interventions, using a validated scoring system.

**Ethics and dissemination:**

Despite widespread use of speech therapy in patients with EILO, this approach is insufficiently tested in RCTs, and the study is therefore considered ethically appropriate. The study will provide knowledge listed as a priority in a recent statement issued by major respiratory and laryngological societies and requested by clinicians and researchers engaged in this area. The results will be presented at relevant conferences, patient fora, and media platforms, and published in relevant peer reviewed international journals.

## Introduction

The larynx represents a narrow and complex valve that controls access to the lower airways and modulates large proportions of total airway resistance during breathing. The larynx serves critical and partly opposing bodily functions, such as maintaining maximal opening during exercise to facilitate high volume ventilation, closing tightly while eating to prevent aspiration, and performing fine-tuned movements during phonation (1–3). The larynx also plays a fundamental role during cough and clearance of secretions (4–6). The understanding of how the larynx influences breathing during exercise in health and disease is at an early stage.

Breathing problems caused by an inappropriate reversible laryngeal adduction in an otherwise apparently normal larynx, is labelled inducible laryngeal obstruction (ILO) (7). When exercise is the inducer of ILO, the acronym EILO is commonly used (8, 9). EILO has previously been labelled in a variety of ways, often using the phrases paradoxical vocal cord motion or vocal cord dysfunction (7). Prevalence rates of 5%–7% are reported in unselected adolescent populations (10, 11), and even higher in groups where exercise is particularly important (12, 13).

**Figure 1:**
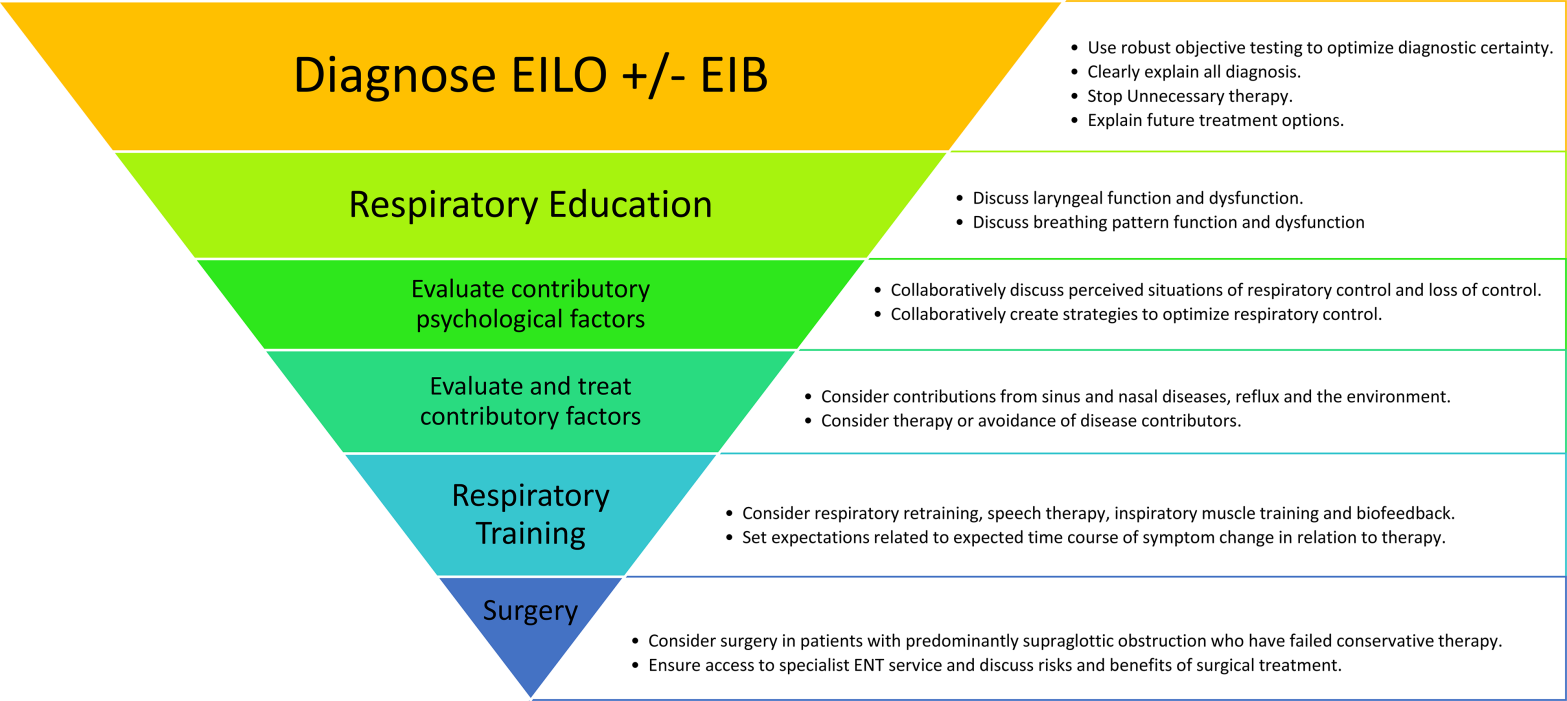
Treatment algorithm for EILO (8).

Studies indicate that EILO responds to treatment interventions; however, we lack randomized controlled studies to confirm this (9, 14–18). Treatment algorithms for EILO generally start with a proper diagnose followed by some form of respiratory education as suggested by Figure 1. Non-surgical treatment includes strategies such as breathing advice (19), speech therapy (20, 21), biofeedback (22, 23), inspiratory muscle training (IMT) (16, 24), laryngeal control therapy (21, 25) and pharmacological treatment (26). Speech therapy has traditionally been considered mainstay therapy for inducible laryngeal obstruction (ILO), with studies reporting symptom resolution or improvement in as many as 89% of patients (27). Searching the literature, it becomes evident that speech therapy in this context has been performed in different ways and according to a variety of protocols. However, the methods applied are often insufficiently described, and therefore difficult to reproduce by others (28). Additionally, treatment is often customised to individual patients, further complicating a search for methods with a more universal application (29). The speech therapy program used at our hospital to treat patients with EILO during the past years has been standardized to be applied in a prospective randomized controlled trial (RCT) called HelpILO (30). The program rests on general speech therapy principles combined with experience obtained with these patients during the past decades. We acknowledge there are several possible approaches to speech therapy in this context. Additionally, we acknowledge recent literature suggesting that EILO comprises at least two distinct categories (glottic and supraglottic) likely to respond differently to speech therapy, which will nonetheless be administered uniformly to all patients.

The aim of the HelpILO study is to test effects of different treatment approaches to EILO in a randomized controlled design in patients consecutively diagnosed with EILO. This article provides in-depth information on the speech therapy protocol used in this study.

## Methods

### HelpILO-study—a randomized controlled treatment trial for patients with EILO

**Figure 2:**
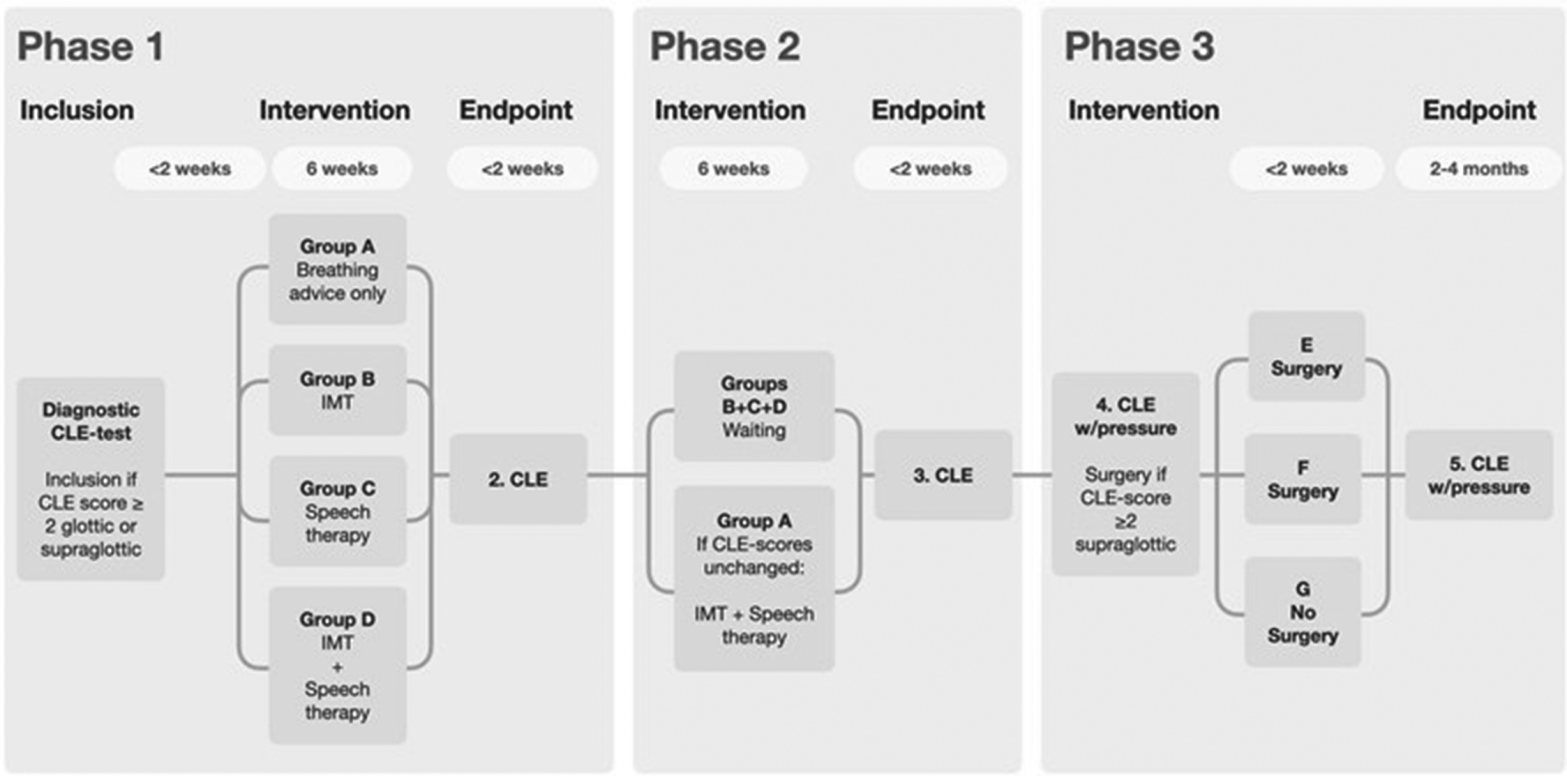
Flow chart of HelpILO (8).

The speech therapy protocol described in this article is part of the HelpILO-study conducted at Haukeland University Hospital during 2021–2024. The study tests commonly used treatment approaches to EILO in a randomized controlled design, enrolling patients consecutively diagnosed with EILO at our institution (Figure 2). Speech therapy constitutes one element of this set-up, in addition to inspiratory muscle training, and basic information and breathing advice guided by visual biofeedback. To assess changes in the larynx, video recordings obtained during exercise before and after interventions will be analysed. The HelpILO study is ethically approved by the Regional Committee for Medical and Health Research Ethics (REK 2020/134444). All participants will provide written informed consent before enrolment.

### EILO diagnostics and grading of outcomes

**Figure 3:**
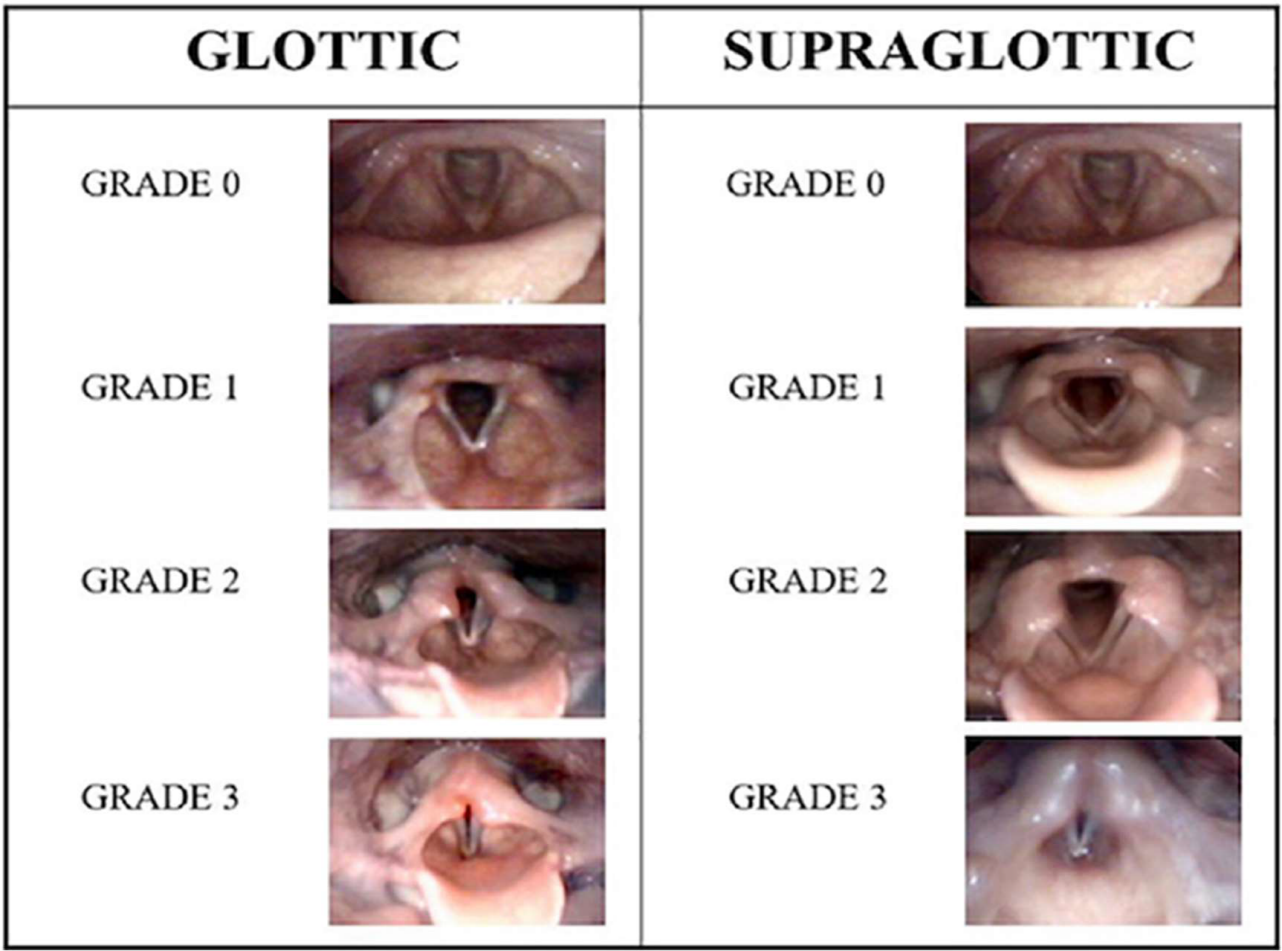
The CLE-scoring system. Reproduced with permission from Fretheim-Kelly et al. (32).

EILO is diagnosed by continuous laryngoscopy performed during exercise (CLE-test) from rest to peak exercise (7, 9, 31). The CLE-test provides visualization of the level of obstruction within the larynx (i.e., glottic, supraglottic or both), and permits timing of the events (i.e., what structures start the adduction, when it occurs, and in what sequence). Evaluation of the laryngeal obstruction, the CLE-score, is based on a categorical scale from 0 to 3 assessing the relative degree of inspiratory adduction of the supraglottic and vocal folds during increasing exercise from a resting condition via moderate to peak exercise (Figure 3) (33). Studies addressing the validity of this CLE scoring system has reached somewhat variable conclusions (33–35). Although the CLE scoring system has its shortcomings, it is to date the most objective measure of EILO severity, it is regularly used in everyday clinic worldwide, and will be used in this present study.

### Developing a speech therapy treatment protocol for EILO over a decade

When speech therapy is used to treat exercise related breathing problems, a range of approaches and manoeuvres can be applied, and the treatment is often tailored to individual patients. At our institution, a treatment protocol for this purpose has slowly been developed during the last 10 years by our speak and language therapist. In order to be transparent and reproducible in this present HelpILO RTC, we have standardized our protocol so that the set of manoeuvres used can be reproduced in a verifiable way also in groups of patients outside our own environment.

The speech therapy applied to treat EILO in the HelpILO study is founded on general principles used in the treatment of voice problems, laryngeal reflexes, and respiratory problems. It focuses on reprogramming involved muscles, and promoting abdominal breathing, with the ultimate goal to facilitate free airflow through the larynx at high volume ventilation induced by exercise. While preparing the treatment strategy, we have emphasized finding approaches that utilize laryngeal reflexes to promote laryngeal patency during inspiration. We therefore tested several manoeuvres while at the same time performing flexible nasal laryngoscopy. This work was commenced in 2009 by our speech therapist (TK) together with two of the co-authors of this article (ODR and JHH). A flexible laryngoscope was used to assess the laryngeal responses to all manoeuvres used in the protocol.

The evolution of our protocol has progressed through four distinct phases over the past decade:

*Phase 1.* The speech therapist (TK) examined different manoeuvres/techniques and assessed the laryngeal responses by flexible laryngoscope of his own larynx.
*Phase 2.* We originally tested four different manoeuvres in 21 healthy students in relation to a speech therapy master thesis, which regrettably is not available online. The four manoeuvres explored were (1) “sniff”, where fast inspiration through the nose was applied to make the larynx open, utilizing a reflex (36) (2) “sob”, loosely based on the “Estill approach”, where the aim is to lower the larynx in the throat to expand the supraglottic area (37), (3) “breathe around the tongue with pursed lips”, a technique that slows down laryngeal airflow during inspiration, thereby creating less laryngeal strain which potentially might reduce the risk of laryngeal adduction or collapse, and (4) “Karlsen's manoeuvre (KM)” where the test-subject inhales through nearly closed teeth with expanded corners of the mouth, a technique that also slows down laryngeal airflow during inspiration, potentially with similar positive effects on laryngeal patency. All participants performed the listed manoeuvres during video laryngoscopy at rest, and four participants additionally performed the manoeuvres during a CLE-test. All participants retrospectively rated the degree of complexity of the manoeuvres and gave feedback on the instructions provided. The video recordings were then evaluated in a blinded randomized fashion by experienced raters (ODR and JHH) to see which manoeuvres efficiently opened up the larynx. Based on this evaluation, the manoeuvres “sob” and “breath around tongue with pursed lips” were rejected, due to both complexity and failure to open the larynx during inspiration as intended.

Additionally, during this preliminary work, two new manoeuvres were included, tested and found suitable in terms of the laryngeal response and patients' assessment of their feasibility. These were “yawning” and “dew-breathing-on-a-imaginary mirror” based on the effect of laryngeal responses observed. Thus, finally there were four manoeuvres found to be suitable and feasible for our purpose: sniff, KM, yawning, and dew-breathing.

Further, aiming for a fully comprehensive treatment approach focused on being mindful of the opening and closing of the larynx, a widely used speech therapy maneuver called “*semi occluded vocal tract voice training and therapy*” was included in the program, using a bottle filled with water and a tube. To increase awareness of the larynx, patients are asked to produce a long “F-sound” during exhalation (later referred to as “f-out”), followed by one of the inspiratory maneuvers. Changes between the maneuvers within one breathing cycle is used to obtain better control over the larynx. Further, actively producing “stridor-like” sounds while inhaling is used to raise the patient's awareness regarding abduction vs. adduction of the larynx.

*Phase 3.* The four manoeuvres (sniff, KM, yawning, and dew-breathing) were used for several years by our speech therapist to treat EILO at our institution. Aiming to test the feasibility of a standardized training approach, seven patients (females, 16–19 year of age) with verified EILO tested a preset program using these maneuvers. The program was based on one visit at the hospital once a week over a 6-week period, supervised by our speech therapist (TK), and additionally practice at home three times daily in between. This was followed by 6 weeks of implementing the techniques during physical activity, while slowly increasing the exercise load.
*Phase 4.* Due to long travel distances for some patients, it became apparent that a more time-intensive protocol was required. Therefore, an alternative more condensed approach was developed to be completed within 2–3 days. Ten patients with verified EILO (age 15–29 years, two males) tested this time-intensive approach under the supervision of our speech therapist (TK), with alternating instructions and self-training. This was followed by 4 weeks of practicing the techniques at home three times daily and gradually implementing the techniques in physical activities. All patient reported the degree of self-training. We observed in this phase that some patients engaged in self-training more frequently, suggesting that this could lead to better results. Patients provided continuous feedback on the treatment and asked questions that were implemented into the patient education, as well as information important to include in the anamneses. Anecdotally, improvements in EILO symptoms were observed with both approaches (phase 3 and phase 4), in line with findings from a similar study (38).

Eventually, five manoeuvres were found suitable for the HelpILO study: (a) the “sniffing” manoeuvre where fast inspiration through the nose is applied to make the larynx open as a reflex (36), (b) a manoeuvre that we have labelled the “Karlsen's manoeuvre” (KM)” where the patient inhales through closed teeth with extended corners of the mouth, which is a technique meant to slow down airflow during inspiration, shown to have a positive effect on laryngeal patency, (c) the yawning manoeuvre, to focus on stretching and widening of the larynx, (d) the dew-breathing manoeuvre (both in and out) to focus on controlling the opening of the larynx, and (e) the “semi occluded vocal tract voice training and therapy” manoeuvre, a well-known technique to “warm-up” the vocal folds. While practicing these techniques, patients are asked to produce a long (39) “F-sound” during exhalation (later referred to as “f-out”), followed by one of the five inspiratory maneuvers. Changes between the maneuvers within one breath cycle is also used to obtain better control. Finally, actively producing “stridor-like” sounds while inhaling is used to raise the patient's awareness regarding abduction vs. adduction of the larynx.

### The speech therapy approach as it is applied in the HelpILO-study

As the HelpILO-study follows an RCT design, the complete speech therapy program had to be standardized and similar in all participating patients. In the beginning of “COVID-pandemic”, we were forced to do most of the treatment supervision by means of online guidance at our EILO-outpatient clinic. Based on our clinical experience (with patients not included in the HelpILO study), we found that the results from online guidance did not clinically differ from the results obtained in patients guided face-to-face. Online guidance therefore became the routine set-up in the HelpILO study.

The speech therapy program in the HelpILO study starts with providing the patient with information about the pathophysiology of EILO, as understood from the point of view of a speech therapist, with explanations supported by figures showing the anatomy of the larynx. The basic principles of an appropriate breathing pattern is explained (3). Thereafter, the patient is guided through a standard exercise program for abdominal breathing, how to obtain and maintain a good posture with a straight back, low shoulders and keeping the head high. The patient is also guided through general principles of how to release tensions in the larynx (40). Abdominal breathing is trained by asking the patient to actively contract the abdominal muscles while exhaling through pursed lips for resistance and then to relax the abdominal muscles and widening the thorax while inhaling (Supplementary Video S1).

Further, the program consists of 5 steps performed during 5 weeks with increasing complexity at each step to maintain progression. Each step lasts 1 week and includes among other elements practicing the five different maneuvers listed in the previous paragraph. The maneuvers are performed in sessions of 15 breaths that are repeated 5–8 times. The breathing exercises at each new step are presented to the patient once a week in a 1 h supervised session under guidance by the speech therapist. Thereafter, the patient is expected to practice individually at home. The last step includes implementing the exercises in physical activities. Progression from one step to the next is determined by the patient's ability to maintain control at the previous step despite the shift in focus that occurs when introducing new elements.

### The five steps used in our speech therapy protocol for EILO

Equipment used in this protocol is a silicone tube, 35 cm long with 12/16 mm inner/outer diameter. In addition to a plastic bottle of 5 dl filled with approximately 3 dl of water. It is emphasized that patients should not use these maneuvers to treat themselves without prior guidance by an experienced therapist (Table 1 for overview).

**Table 1:** Schematic overview of the treatment.

Appointment	Contents	Exercise
1	0	1. Anamnesis/information 2. Patient education 3. Abdominal breathing by exercises 1 and 2
	1	Semi occluded vocal tract voice training and therapy with water, bottle and tube
	2	Breathing “f-out^a^” and yawn in
2	1	Semi occluded vocal tract voice training and therapy with water, bottle, and tube
	2	Breathing “f-out” and yawn in
	3	F-out, sniff in
	4	F-out, KM^b^ in
	5	Dew out, dew in
3	1	Semi occluded vocal tract voice training and therapy with water, bottle and tube
	2	F-out, stridor in; f-out, yawn in
	3	F-out, stridor in; f, out, sniffing in
	4	F-out, stridor in; f-out, KM in
	5	F-out, stridor in; dew-out, dew-in
4	1	Semi occluded vocal tract voice training and therapy with water, bottle and tube
	2	F-out, stridor in; start by inhaling stridor in, then terminate by inhaling sniffing in
	3	F-out, stridor in; start by inhaling stridor in, then terminate by inhaling KM in
	4	F-out, stridor in; start by inhaling stridor in, then terminate by inhaling yawn in
5	0	Implement and automate the techniques in physical activity. Check once a month
	1	Semi occluded vocal tract voice training and therapy with water, bottle and tube
	2	Breathing “f-out” and yawn in
	3	F-out, KM in

^a^ F-out: the labiodental/f/fricative in exhalation, as of Friday. ^b^KM: Karlsen's maneuver, inhaling through closed teeth with extended corners of the mouth.

Step 1:
  1. The first exercise focuses on the “*semi occluded vocal tract voice training and therapy*” maneuver where he/she use a wide tube to blow into a bottle of water (39). This generates a positive airway pressure, widening the larynx during expiration (Supplementary Video S2).
  2. Breathing “*f-out*” *and yawn in,* i.e., inhaling throughout the yawn (Supplementary Video S3).
Step 2:

This step focuses on the previous two exercises in addition to three new ones.

1. “*Semi occluded vocal tract voice training and therapy*”
2. “*F-out, yawn in*”
3. “*F-out, sniff in*”*.* Inhalation by sniffing in through the nose (Supplementary Video S4).
4. “*F-out, KM in*” (Supplementary Video S5).
5. “*Dew out, dew in*”. Inhaling without making any changes on the articulatory position (Supplementary Video S6).

Step 3:

The objective of this step is to make the patient aware of the feeling regarding abduction vs. adduction of the larynx. The following maneuvers are used in the training:

1. “Semi occluded vocal tract voice training and therapy”
2. “*F-out, stridor in*”*;* “*f-out, yawn in*” (Supplementary Video S7).
3. “*F-out, stridor in*”*;* “*f, out, sniffing in*” (Supplementary Video S8).
4. “*F-out, stridor in*”*;* “*f-out, KM in*” (Supplementary Video S9).
5. “*F-out, stridor in*”*;* “*dew-out, dew-in*” (Supplementary Video S10).

The patient will focus on flow and execution first. When performance is sufficient, the patient focuses on the contrasts in the sensation of stridor and opening of the larynx.

Step 4:

The objective of this step is to use the techniques to stop an attack of EILO. The experiences from Step 3 are reviewed. This step will focus on making “stridor-like noise” and then subsequently changes to making no noise, using the techniques practiced in the prior sessions:

1. “*Semi occluded vocal tract voice training and therapy*”
2. F-out, stridor in; start by inhaling stridor in, then terminate by inhaling “sniffing in” (Supplementary Video S11).
3. F-out, stridor in; start by inhaling stridor in, then terminate by inhaling “KM in” (Supplementary Video S12).
4. F-out, stridor in; start by inhaling stridor in, then terminate by inhaling “yawn in” (Supplementary Video S13).

Step 5:

The objective of this step is to start implementing the maneuvers during physical activity. The patient is informed to start practicing the different breathing techniques while performing low to moderate intensity exercise and gradually increase the intensity while maintaining abdominal breathing and laryngeal control. It is the sense of breathing control that should determine the degree of physical activity. The aim is to help the patient develop a strategy on how to control his/her larynx during exercise, and to be able to continue exercising without experiencing EILO incidents. It is emphasized that the adopted breathing techniques need to be repeated until they become adapted as a part of their automated breathing pattern.

### Statistical methods

The HelpILO-study has been described in detail in a previous communication, also encompassing power calculations to ensure that the planned number of enrolled participants (*N* = 350) will be sufficient to detect clinically significant effects (30). Speech therapy will be compared to simple information and breathing advice and inspiratory muscle training. Sub-analyses will be performed to test if the treatments execute differently in glottic vs. supraglottic EILO, as suggested previously for inspiratory muscle training in an uncontrolled study (19). As secondary outcomes, participants will fill in standardized questionnaires before and after treatment, to evaluate symptom scores.

## Discussion and summary

Given the high prevalence of EILO, we are concerned by the limited understanding of the role played by the larynx during exercise and the lack of evidence-based treatment approaches. We here present our speech therapy treatment protocol for EILO, which constitutes an important element in the large prospective randomized controlled HelpILO study. This represents a first attempt to generate structured speech therapy treatment algorithms for patients with EILO. Based on the results, we hope to be able to generate more scientifically robust treatment algorithms, and informed choices as regards treatment of EILO. Speech and language therapy represents a comprehensive profession, and the tools used vary by tradition between institutions and countries (41). We acknowledge that there are various approaches that could be feasible and effective for the treatment of EILO, with notable examples including the EILOBI technique (42) and the treatment for breathing pattern disorder recently described by Milstein et al. (43). The protocol used in this present study is based on a set of maneuvers which are expected to have the desired effect on the patients' breathing problem. Thus, we cannot distinguish if specific maneuvers or treatment elements is more important than others in individual patients.

Further, EILO is probably not a distinct phenotype, but likely to consist of subgroups characterized by different pathophysiology which probably also respond differently to treatment (16, 44). Moreover, patients with EILO differ as regards age, gender, comorbidities, level of physical activity, expectations, and ambitions in relation to physical performance, as well as motivation and compliance with treatment, aspects that are bound to influence the efficacy of the treatment offered by health care providers. In the HelpILO study, all enrolled patients will be treated similarly, opening for sub-grouping by treatment responses. To obtain these goals, we had to approach speech therapy in a standardized and non-flexible way, applying maneuvers which we empirically have found feasible and effective in clinics.
